# Glypican-1 regulates myoblast response to HGF via Met in a lipid raft-dependent mechanism: effect on migration of skeletal muscle precursor cells

**DOI:** 10.1186/2044-5040-4-5

**Published:** 2014-02-12

**Authors:** Jaime Gutiérrez, Daniel Cabrera, Enrique Brandan

**Affiliations:** 1 Centro de Regulación Celular y Patología (CRCP), Centro de Regeneración y Envejecimiento (CARE), Departamento de Biología Celular y Molecular, MIFAB, Pontificia Universidad Católica de Chile, Santiago, Chile

**Keywords:** Glypican-1, Heparan sulfate proteoglycans, Hepatocyte growth factor, HGF-mediated signaling, Raft membrane domains, Skeletal muscle

## Abstract

**Background:**

Via the hepatocyte growth factor receptor (Met), hepatocyte growth factor (HGF) exerts key roles involving skeletal muscle development and regeneration. Heparan sulfate proteoglycans (HSPGs) are critical modulators of HGF activity, but the role of specific HSPGs in HGF regulation is poorly understood. Glypican-1 is the only HSPG expressed in myoblasts that localize in lipid raft membrane domains, controlling cell responses to extracellular stimuli. We determined if glypican-1 in these domains is necessary to stabilize the HGF-Met signaling complex and myoblast response to HGF.

**Methods:**

C2C12 myoblasts and a derived clone (C6) with low glypican-1 expression were used as an experimental model. The activation of Met, ERK1/2 and AKT in response to HGF was evaluated. The distribution of Met and its activated form in lipid raft domains, as well as its dependence on glypican-1, were characterized by sucrose density gradient fractionation in both cell types. Rescue experiments reexpressing glypican-1 or a chimeric glypican-1 fused to the transmembrane and cytoplasmic domains of mouse syndecan-1 or myoblast pretreatment with MβCD were conducted. *In vitro* and *in vivo* myoblast migration assays in response to HGF were also performed.

**Results:**

Glypican-1 localization in membrane raft domains was required for a maximum cell response to HGF. It stabilized Met and HGF in lipid raft domains, forming a signaling complex where the active phospho-Met receptor was concentrated. Glypican-1 also stabilized CD44 in a HGF-dependent manner. In addition, glypican-1 was required for *in vitro* and *in vivo* HGF-dependent myoblast migration.

**Conclusions:**

Glypican-1 is a regulator of HGF-dependent signaling via Met in lipid raft domains.

## Background

The process of skeletal muscle regeneration is initiated immediately after injury by the release of growth factors and cytokines from injured muscles, blood vessels, infiltrating inflammatory cells and extracellular matrix (ECM) reservoirs. These factors include basic fibroblast growth factor 2 (FGF-2) and hepatocyte growth factor (HGF) [1-3]. The factors promote the activation, proliferation, migration and survival of satellite cells (SCs), which are the muscle stem cells responsible for the formation of new muscle fibers [2]. HGF was originally identified as a scatter factor because of its ability to increase the motility of several normal and neoplastic cells [4,5]. The requirement of HGF for migration of muscle precursor cells during mouse muscle development has been established by the genetic ablation of HGF or the HGF receptor (Met). In both cases, the result was the absence of hindlimb muscles, which are formed by muscle precursor cells that migrate from the dermomyotome [6-8]. *In vitro* studies have shown that HGF not only induces the proliferation and migration of myogenic cells but that it also delays muscle differentiation by inhibiting the expression of MyoD and myogenin, two master myogenic regulatory transcription factors [3,9,10]. The expression of HGF and Met are downregulated during myogenesis, which is consistent with attenuation of myogenic inhibitory signaling of HGF [11-13]. Therefore, HGF plays key role during myogenesis, regulating the proliferation, migration and subsequent differentiation of muscle precursor cells.

Upon HGF binding, Met is activated by dimerization with subsequent *trans*-phosphorylation of four tyrosine residues which act as docking motifs for signaling mediators, including mitogen-activated protein kinase (MAPK), extracellular signal-regulated kinases 1 and 2 (ERK1/2) and phosphoinositide 3-kinase protein kinase B (AKT), among others [14-16].

It has been proposed that HGF and Met form a complex in lipid rafts, which are sphingolipid- and cholesterol-rich domains that form phase-separated lipid rafts in the membrane. In these domains, Met is stabilized by HGF to induce its activation [17-20].

Another important component of the HFG-Met signaling is the ubiquitous transmembrane glycoprotein CD44, the major receptor for hyaluronic acid [21,22]. In different cell types, the activation of the MET receptor by HGF depends on the presence of some isoforms of CD44 [21]. As proposed, HGF, Met and CD44 would form a complex in lipid raft membrane domains, which corresponds to sphingolipid- and cholesterol-rich domains forming phase-separated lipid rafts in the membrane, where Met would be stabilized by HGF inducing its activation [17,18].

HGF also binds to heparin, heparan sulfate (HS) and dermatan sulfate [23-27]. Heparan sulfate proteoglycans (HSPGs), key components of the cell surface and the ECM, regulate many processes related to cell growth and differentiation. Cell-surface HSPGs bind soluble ligands, increasing their local concentration and modulating ligand–receptor interactions [28]. For example, HSPG is required for FGF-2-dependent signaling through its receptors (FGFRs) [29-32], forming the ternary complex HSPG-FGF-2-FGFR [33]. However, the exact role of HSPG in HGF signaling is poorly understood. *In vitro* assays have shown that heparin increases the mitogenic effect of HGF and facilitates its oligomerization, inducing Met dimerization and activation [34]. Previously, we showed that myoblast migration induced by HGF was strongly inhibited if the cells were depleted of HS chains, indicating that at least the myoblast cell response to HGF depended on HS [23].

We have also previously shown that myoblasts express different membrane-bound HSPGs, the four transmembrane syndecans and glypican-1, which corresponds to a glycosylphosphatidylinositol-anchored HSPG [31,32,35-39]. Glypican-1 is the only HSPG located in lipid raft microdomains, which sequester FGF-2 to avoid its interaction with FGFRs. Thus, glypican-1-deficient cells exhibit enhanced sensitivity to FGF-2. In contrast, HGF-dependent signaling was clearly decreased in the absence of glypican-1, suggesting that glypican-1 was a positive regulator of HGF signaling [38].

Because HGF and Met are found in lipid raft domains [17-20], we hypothesize that glypican-1 in these domains is necessary to stabilize the HGF-Met signaling complex. In the present study, we report that the presence of glypican-1 in lipid rafts was required for maximum HGF-dependent signaling, localizing and stabilizing HGF and Met in its phosphorylated or activated state (phospho-Met). We also show that glypican-1, phospho-Met and HGF interact, indicating that they form part of a signaling complex in lipid rafts. Finally, we show that glypican-1 is required for myoblast migration induced by HGF *in vitro* and *in vivo*, demonstrating the requirement of glypican-1 expression and HGF for processes such as muscle stem cell therapy, where the migration of myoblasts must be enhanced.

## Methods

### Cell culture

The mouse skeletal muscle cell line C2C12 (American Type Culture Collection, Manassas, VA, USA) [40] and its derived clone deficient in glypican-1 expression [38] were grown as previously described [31,38]. Myoblasts were treated with HGF (R&D Systems, Minneapolis, MN, USA) as indicated in each experiment. Methyl-β-cyclodextrin (MβCD) (Sigma-Aldrich, St Louis, MO, USA) treatment at 1 or 10 mM concentrations were performed as previously described [38]. For the phosphorylation experiments of Met, ERK1/2 and AKT, the cells were serum-starved for 6 hours and then treated for the indicated times.

### Transient transfection and generation of stable clones

The pcDNA3.0 empty vector (Invitrogen, Carlsbad, CA, USA) and pcDNA3.0 vectors containing rat glypican-1 and chimeric HSPG comprising the extracellular domain of rat glypican-1 were fused to the transmembrane and cytoplasmic domains of mouse syndecan-1 containing a FLAG epitope in their amino-terminal F-Gly and F-GlySyn, respectively [38]. Transfection were carried out using Lipofectamine and PLUS reagents (Invitrogen) according to the supplier’s protocol.

### Isolation of lipid rafts

Lipid rafts were prepared as described previously, with some modifications [38]. All of the buffers and instruments used in the procedure described below were used at 4°C. Briefly, C2C12 myoblasts from a 150-mm dish were lysed in 400 μl of lysis buffer (25 mM 2-(*N-*morpholino)ethanesulfonic acid, pH 6.5, 150 mM NaCl, with a mixture of protease inhibitors and 1 mM phenylmethanesulfonyl fluoride supplemented with 1% Triton X-100). Cells were incubated for 20 minutes on ice, then homogenized with ten strokes of a loose-fitting Dounce homogenizer. Homogenates were mixed with 400 μl of 90% sucrose (45% final concentration), loaded at the bottom of a Sorvall 4-ml centrifuge tube (Thermo Scientific, Asheville, NC, USA) and overlaid with 1.6 ml of 35% sucrose and 1.6 ml of 5% sucrose, both in the lysis buffer without Triton X-100. The samples were centrifuged at 45,000 rpm for 18 hours at 4°C in an AH-650 rotor. Twelve fractions (330 μl each) were collected from top to bottom and designated as fractions 1 to 12. Only the last ten fraction were analyzed, because the low-density lipid raft–enriched fractions started at fraction 5 in several previous assays that we performed.

### SDS-PAGE, Western blot and coimmunoprecipitation assays

Aliquots from the last ten fractions of the different sucrose density fractionations were separated on 8% SDS-PAGE gels (Mini-PROTEAN II; Bio-Rad Laboratories, Hercules, CA, USA) and electrophoretically transferred to Immobilon membranes (EMD Millipore, Bedford, MA, USA). Western blots were probed using the following primary antibodies: rabbit anti-mouse Met (1:200) (Santa Cruz Biotechnology, Santa Cruz, CA, USA), rabbit anti-phospho-Met at Tyr 1234 and Tyr 1235 (1:1,000) (Cell Signaling Technology, Danvers, MA, USA), rabbit anti-caveolin-1 (1:500) (Santa Cruz Biotechnology), rabbit anti-glypican-1 M-95 (1:500) (Santa Cruz Biotechnology), mouse anti-Na^+^/K^+^-ATPase (1:1,000) (Upstate Biotechnology, Lake Placid, NY, USA) and rat anti-CD44 (1:500) (BD Pharmingen, San Jose, CA, USA).

To identify glypican-1, samples containing equivalent amounts of protein were treated with heparitinase and chondroitinase ABC (United States Biological, Swampscott, MA, USA) as previously described [39,41] prior to SDS-PAGE and Western blot analysis using anti-glypican-1 M-95 antibody.

For analysis of phosphorylated proteins, cell extracts were prepared in radioimmunoprecipitation assay (RIPA) buffer in the presence of phosphatase inhibitors as previously described [38,42]. Aliquots with equivalent amounts of protein were subjected to SDS-PAGE in 8% polyacrylamide gels, electrophoretically transferred to Immobilon membranes (EMD Millipore) and probed with the following antibodies: rabbit anti-phospho-ERK1/2 (1:1,000), mouse anti-FLAG (1:5,000) (Stratagene, La Jolla, CA, USA), rabbit anti-ERK1/2 (1:1,000), rabbit anti-phospho-AKT (1:1,000) (Calbiochem, San Diego, CA, USA), mouse anti-α-tubulin (1:5,000) (Sigma-Aldrich), mouse anti-myosin (1:5,000) (Sigma-Aldrich) and mouse anti-glyceraldehyde 3-phosphate dehydrogenase (1:2,000) (Chemicon International, Temecula, CA, USA). All immunoreactions were visualized by enhanced chemiluminescence (Pierce Biotechnology, Rockford, IL, USA) using a ChemiDoc-It 410 high-resolution imaging system (UVP, Upland, CA, USA).

For coimmunoprecipitation experiments, wild-type and glypican-1-deficient myoblasts (C6) were transiently transfected as indicated in the figure legends. At 48 hours after transfection, cells were serum-starved for 4 hours, then either treated or not treated with 20 ng/ml [^125^I]HGF in Dulbecco’s modified Eagle’s medium (DMEM) 0.1% bovine serum albumin (BSA) for 5 minutes. The cell extracts in RIPA buffer plus phosphatase inhibitors were incubated with anti-FLAG M2 Affinity Gel (Sigma-Aldrich) for 3 hours at 4°C. The beads were sequentially washed in RIPA buffer, then in heparitinase reaction buffer (20 mM Tris, 150 mM NaCl, 1 mM MgCl_2_, 1 mM CaCl_2_, pH 7.4). The beads were then treated with heparitinase and chondroitinase ABC for 3 hours at 37°C. The bound material was eluted with protein loading buffer and assessed by Western blot analysis for total Met, phospho-Met and glypican-1 or exposed to a phosphorimager to detect [^125^I]HGF.

### Biotin labeling and precipitation of biotin-labeled proteins

Biotin labeling was conducted as previously described [43]. Equal amounts of protein (100 μg) obtained from previously biotinylated cell extracts were precipitated for 2 hours at 4°C using streptavidin agarose resin (Thermo Fisher Scientific, Rockford, IL, USA). The bound material was eluted with protein loading buffer and assessed by Western blot analysis for total Met as described above.

### Transwell migration assays

Migration assays were conducted using 24-well, 8-μm-pore transwell systems (EMD Millipore). C2C12 and C6 myoblasts were seeded onto the upper part of the chamber at a density of 100,000 cells per well in 300 μl of serum-free media. The lower chamber was loaded with 500 μl of serum-free media with or without 20 ng/ml HGF or 10% fetal bovine serum (FBS) (data not shown). The cells were allowed to migrate for 8 hours. Migration was assessed by removing the cells on the upper side of the transwell with a cotton swab, then staining the remaining cells with crystal violet, and solubilizing the cells in 1% Triton X-100 to measure the absorbance of the Triton X-100 solution at 595 nm [44].

### *In vivo* myoblast migration assay

Myoblasts were labeled with the vital dialkylcarbocyanine dye DiI (red fluorescence) according to the supplier’s protocol (Sigma-Aldrich). Aliquots containing 500 × 10^3^ myoblasts were resuspended in 30 μl of physiological serum and kept on ice. Immediately before grafting, 1 μl of physiological serum containing or not containing 10 ng of carrier-free HGF was added to myoblast. Three-month-old C57BL/10 mice were used as hosts, and cells were slowly injected longitudinally in both tibialis anterior (TA) muscles of mice under isoflurane gas anesthesia. Cells treated or not treated with HGF were injected into the contralateral TA muscles. After 7 days, the TA muscles were snap-frozen in isopentane before being entirely cut in transversal 7-μm cross-sections. Muscle cross-sections were visualized under a Nikon Diaphot inverted microscope (Nikon Instruments, Melville, NY, USA) equipped for epifluorescence. Concentric rings disposed 200 μm from each other were superimposed on the selected muscle cross-section photographs. The total number of migrating myoblasts was determined by counting the labeled cells that had migrated more than 200 μm from the injection site (which was determined by the border of the more intense fluorescence) [45]. The percentage of cells that reached more than 600 μm over the total migrating myoblast was quantified. These percentages were used to compare the migration of myoblasts between the different conditions. All mice had free access to water and a chow diet until they were studied. All protocols were conducted in strict accordance with the formal approval of the Animal Ethics Committee of the Pontificia Universidad Católica de Chile.

### Hepatocyte growth factor affinity labeling and binding assay

Carrier-free HFG was radiolabeled with Na^125^I using the chloramine T method as previously described for FGF-2 [38]. The biological activity of the radiolabeled HGF was determined by its ability to induce phosphorylation of ERK1/2 compared to unlabeled HGF as described above. The binding of [^125^I]HGF to cell surfaces was performed as described previously with some modifications [46]. Briefly, subconfluent myoblasts were incubated for 2 hours at 4°C in DMEM containing 0.2% BSA, 25 mM 2-[4-(2-hydroxyethyl)piperazin-1-yl]ethanesulfonic acid (HEPES), pH 7.4, and 10 ng/ml [^125^I]HGF. To determine nonspecific binding, parallel cultures were incubated under the same conditions with the addition of a 200-fold excess of unlabeled HGF. After several washes in binding buffer and once with phosphate-buffered saline to remove unbound ligand, the cells were sequentially washed twice with 2 M NaCl in 20 mM HEPES, pH 7.4, for 5 minutes (low affinity binding) and twice with 2 M NaCl in 20 mM NaAc, pH 4.0, for 5 minutes (high-affinity binding) [47-49]. The cells were extracted, and the protein content was determined as indicated below. The amount of radioactivity present in the low- and high-affinity washes and cell extracts was determined using a γ scintillation counter. The counts per minute (cpm) values were corrected for the protein content in the cell extracts.

### Protein determination

Protein content in cell extracts was determined with a bicinchoninic acid protein assay kit (Pierce Biotechnology) with BSA used as the standard according to the supplier’s protocol.

### Statistical analyses

The number of replicates is indicated in the figure legends for each experiment. Data are presented as the mean ± standard deviation. Statistical significance was assessed using two-way analysis of variance and a Bonferroni multiple-comparisons posttest. Differences were considered statistically significant at *P* < 0.05.

## Results

### Myoblasts require glypican-1 expression for proper hepatocyte growth factor signaling

**Figure 1:**
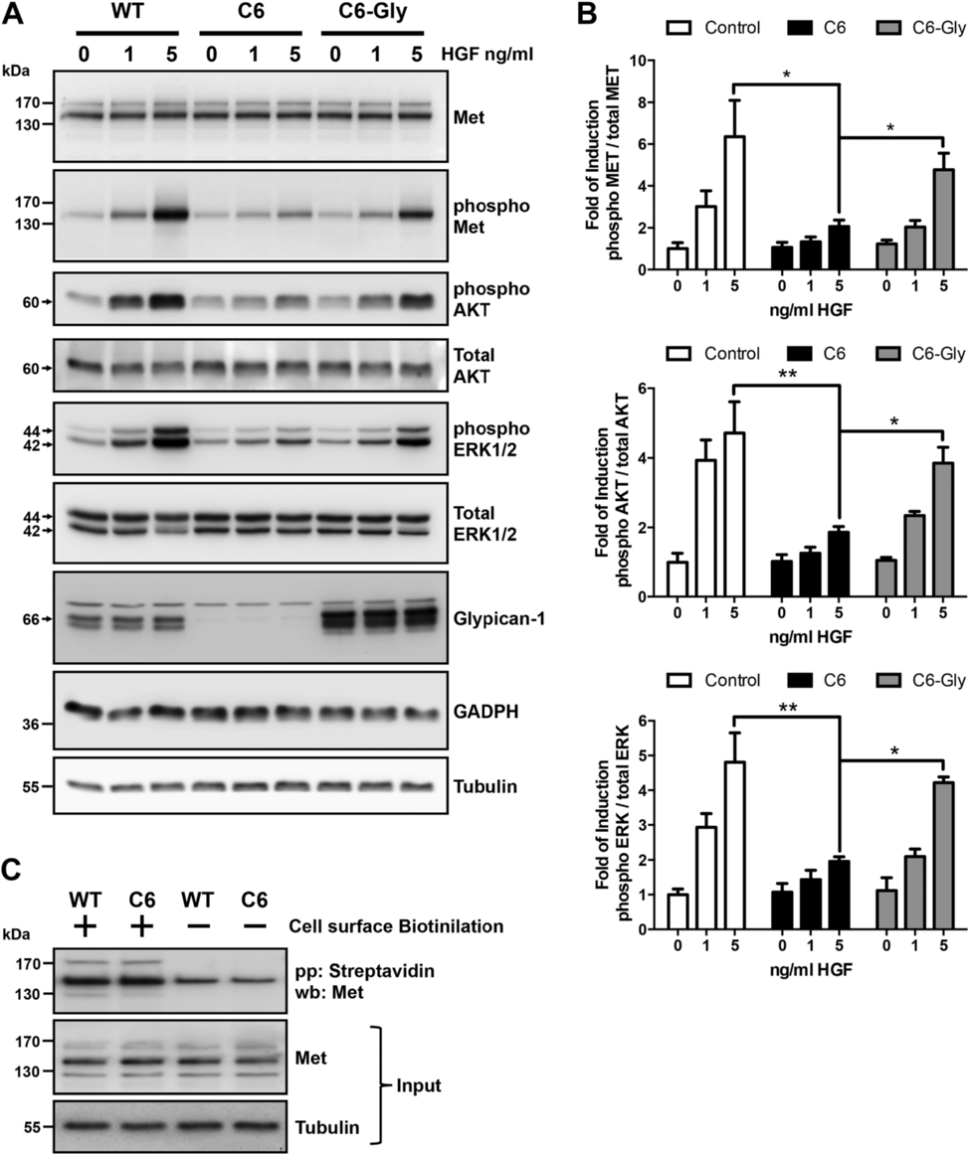
**Myoblasts require glypican-1 expression for proper hepatocyte growth factor signaling. (A)** Wild-type (WT) C2C12 myoblasts and C6 myoblasts (glypican-1-deficient clone) transiently transfected with rat glypican-1 (C6-Gly), were serum-starved for 6 hours and then treated with the indicated concentrations of hepatocyte growth factor (HGF) for 5 minutes. The cell extracts were analyzed by immunoblotting for total HGF receptor (Met) levels, phospho-Met (Tyr 1235/1349), phospho- and total AKT levels, phospho- and total levels of extracellular signal-regulated kinases 1 and 2 (ERK1/2), glypican-1 core protein (after heparitinase treatment), glyceraldehyde 3-phosphate dehydrogenase (GAPDH) and tubulin. Total Met, AKT and ERK1/2 were used as loading control of its respective phosphorylated forms. GADPH and tubulin were used as loading control of glypican-1 expression levels. The Western blot images are representative of three independent experiments. **(B)** Quantitation of phospho-Met, phospho-AKT and phospho-ERK1/2 from three independent experiments is shown. Values are expressed as mean ± standard deviation. Statistical significance was assessed using two-way analysis of variance and a Bonferroni multiple-comparisons posttest. *P < 0.05, **P < 0.01. **(C)** Cell surface proteins of WT and C6 myoblasts labeled with EZ-Link Sulfo-NHS-Biotin (Pierce Biotechnology) as described in Methods. Aliquots of the cell extracts containing equal amounts of protein were precipitated with streptavidin-sepharose beads. The bound material was analyzed by Western blot immunoblotting against total Met. Aliquots of each assay obtained prior to the precipitation were analyzed by Western blot immunoassay for total Met, with tubulin used as the input control. Molecular weight standards are shown at left.

To evaluate the role of glypican-1 in the myoblast response to HGF, C2C12 myoblasts and the derived clone C6, which expresses low levels of HSPG [38], were treated with increasing concentrations of HGF. Phosphorylation of the Met receptor (phospho-Met) and the second messengers AKT (phospho-AKT) and ERK1/2 (phospho-ERK1/2) in response to HGF were analyzed by Western immunoblotting. Figure 1A shows that the phosphorylation levels of Met, AKT and ERK1/2 increased in a HGF concentration–dependent manner. However, glypican-1-deficient myoblasts required higher concentrations of HGF to induce phosphorylation of the same proteins. The diminished response to HGF in the absence of glypican-1 was specific, because glypican-1 reexpression resulted in the rescue of HGF sensitivity. The same figure comparing wild-type (WT), glypican-1-deficient and glypican-1-overexpressing myoblasts also shows that the total levels of Met, AKT and ERK1/2 were not affected by the different conditions of glypican-1 expression. Quantification values from three independent experiments are shown in Figure 1B. Figure 1C shows that expression levels of Met present at the cell surface were unaltered by the presence or absence of glypican-1, as determined by labeling of the extracellular proteins with biotin followed by precipitation with streptavidin-agarose and detection with a specific anti-Met receptor antibody using Western blots.

**Figure 2:**
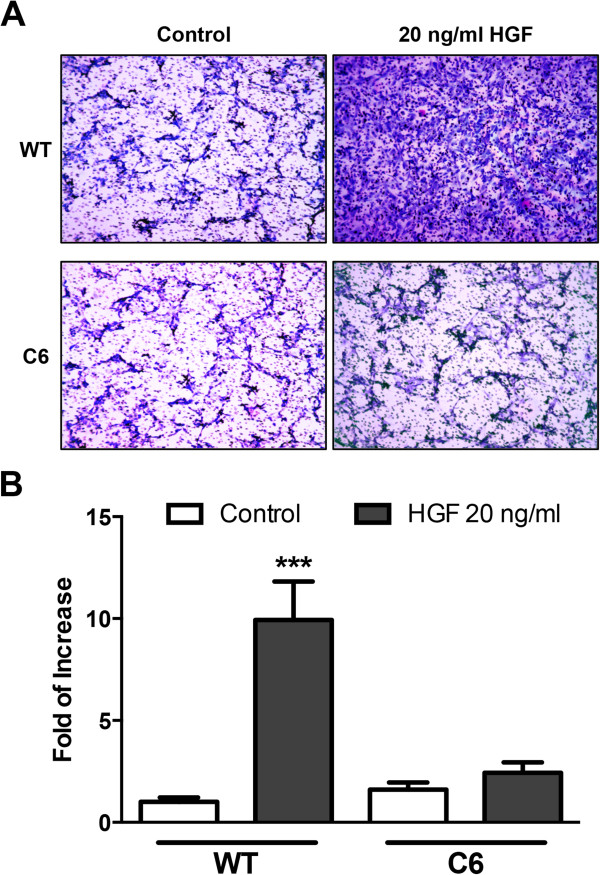
**Hepatocyte growth factor-dependent myoblast migration requires glypican-1 expression. (A)** Wild-type (WT) C2C12 and C6 myoblasts were seeded onto the upper part of transwell chambers at the same density in serum-free media. The lower chamber contained serum-free media with or without 20 ng/ml hepatocyte growth factor (HGF). After 8 hours, the cells in the upper part of the filter were scraped. The cells that had efficiently migrated through the filter were fixed with paraformaldehyde, stained with crystal violet and photographed or as shown in **(B)** stained with crystal violet and solubilized in phosphate-buffered saline containing 1% Triton X-100. The absorbance of the detergent soluble fraction at 595 nm was determined. Values are expressed as mean ± standard deviation of three independent experiments. ***P < 0.001 relative to WT control. The migration of WT under control conditions corresponds to a value of 1.0.

Because muscle precursor cells migrate in response to HGF during skeletal muscle development and regeneration, we decided to evaluate the role of glypican-1 in HGF-dependent migration. Figures 2A and 2B show that HGF induces the migration of WT myoblasts tenfold. In contrast, less than twofold induction was found in glypican-1-deficient myoblasts. In the absence of HGF, WT and glypican-1-deficient myoblast migration was essentially the same. Together, these results suggest that glypican-1 is required for a proper myoblast response to HGF, as determined by activation of HGF-dependent signaling and myoblast migration.

### Met is localized and activated in lipid rafts by a HGF- and glypican-1-dependent mechanism

**Figure 3:**
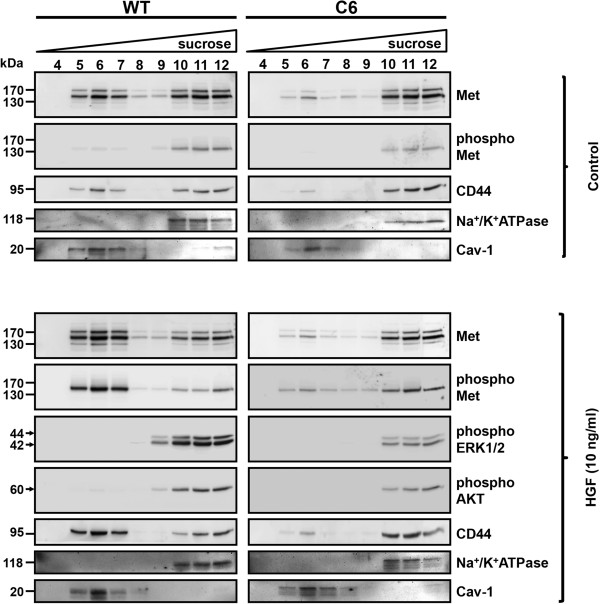
**Met is localized and activated by hepatocyte growth factor in lipid rafts by a glypican-1-dependent mechanism.** C2C12 and C6 myoblasts were serum-starved for 6 hours and then treated without (control) or with 10 ng/ml hepatocyte growth factor (HGF) for 5 minutes. The cells were lysed with 1% Triton X-100 and fractionated by sucrose density gradients (5% to 45%). Twelve fractions were collected, but only the last ten fractions were analyzed (the lipid raft–enriched fraction started at fraction 4) by immunoblotting for total HGF receptor (Met), phospho-Met (Tyr 1235/1349), phosphorylated extracellular signal-regulated kinases 1 and 2 (phospho-ERK1/2), phospho-AKT and CD44. As fractionation controls, the presence of the lipid raft membrane protein marker caveolin 1 (Cav 1) and the non-lipid-raft domain marker Na^+^/K^+^-ATPase are shown. WT, Wild type.

We have shown that glypican-1 was the only HSPG associated with lipid raft microdomains in myoblasts [38]. The results presented in Figures 1 and 2 suggest that glypican-1 acts as a positive regulator of HGF signaling. Therefore, we studied the association of Met with lipid raft membrane domains and the possible role of glypican-1 and HFG in this localization. To accomplish this objective, WT and glypican-1-deficient myoblasts were either untreated or treated with 10 ng/ml HGF, then fractionated in sucrose density gradients. Figure 3 shows that in untreated WT myoblasts (control), Met fractionated in lipid rafts (fractions 5, 6 and 7) and non-lipid-raft fractions (fractions 10, 11 and 12) to almost the same extent. In contrast, in glypican-1-deficient myoblasts, almost all Met fractionated in the non-lipid-raft fractions. In both WT and glypican-1-deficient myoblasts, the basal phosphorylation level of Met (as shown in Figure 1) was exclusively present in non-lipid-raft fractions. The distributions of caveolin 1 and sodium/potassium ATPase (Na^+^/K^+^-ATPase) were used as lipid raft and non-lipid-raft markers, respectively. These results suggest that glypican-1 is required to distribute part of the total HGF receptor to lipid raft domains. After the treatment with HGF, the proportion of total Met in lipid rafts vs. non–lipid rafts was augmented in WT myoblasts, but not in glypican-1-deficient myoblasts. Importantly, in WT myoblasts, most of the phospho-Met was associated with lipid raft fractions. In contrast, in the glypican-1-deficient myoblasts, most of the phospho-Met was associated with non-lipid-raft fractions. In both WT and glypican-1-deficient myoblasts, phospho-ERK1/2 and phospho-AKT were found in the non-lipid-raft fractions.

Next, we evaluated the presence of CD44 in lipid raft domains and its dependence on glypican-1. Our results show that the association of CD44 with the lipid raft domain is dependent on glypican expression and that this association is stabilized after pretreatment with HGF (Figure 3).

**Figure 4:**
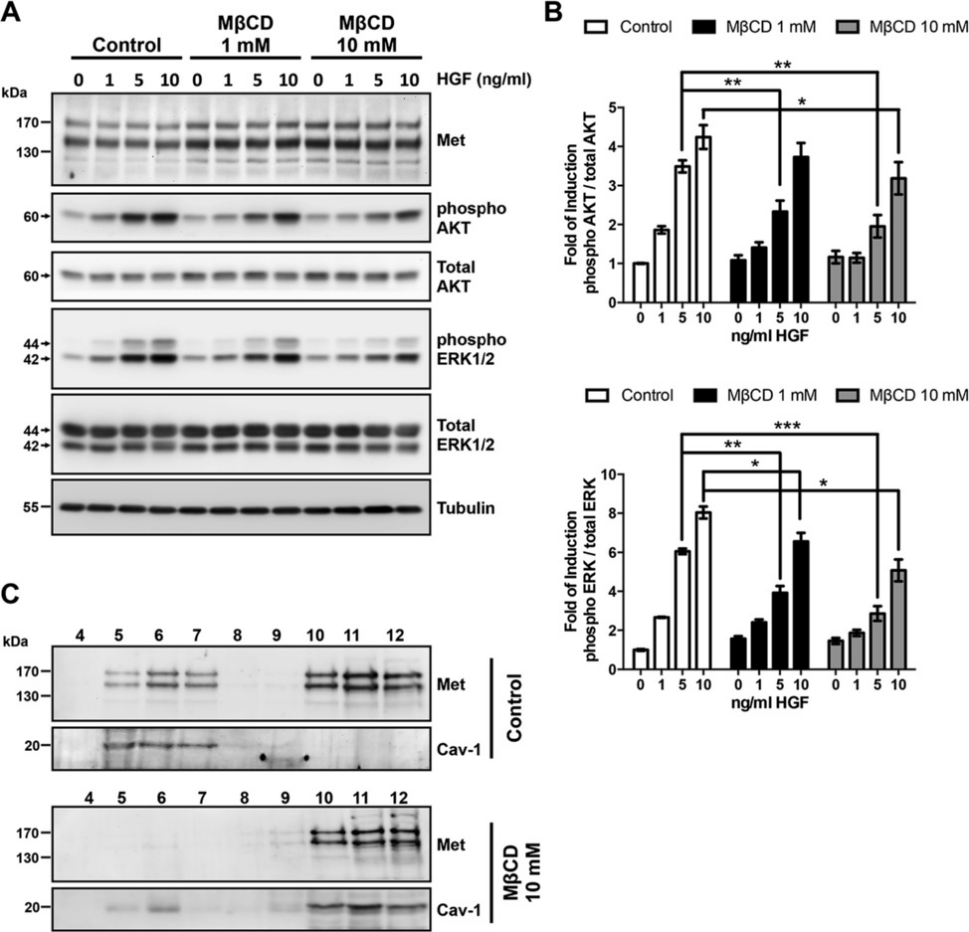
**Disruption of lipid rafts diminishes hepatocyte growth factor–dependent signaling. (A)** C2C12 myoblasts were serum-starved for 6 hours, and during the last hour the cells were treated with or without methyl-β-cyclodextrin (MβCD) at the indicated concentrations. After two washes with serum-free media, the cells were treated with the indicated concentrations of hepatocyte growth factor (HGF) for 5 minutes. The cell extracts were analyzed by immunoblotting for total HGF receptor (Met), phospho- and total AKT, phosphorylated extracellular signal-regulated kinases 1 and 2 (phospho-ERK1/2) and total ERK1/2, and tubulin was used as a loading control. **(B)** Quantification from two independent experiments is shown. Statistical significance was assessed using two-way analysis of variance and a Bonferroni multiple-comparisons posttest. *P < 0.05, **P < 0.01, ***P < 0.001. **(C)** C2C12 myoblasts treated with or without 10 mM MβCD for 1 hour as described in **(A)** were lysed and fractionated in sucrose density gradients as described in Figure 3. The distributions of total Met and caveolin 1 (Cav-1) were determined by immunoblot analysis. In **(A)** and **(C)**, the molecular weight standards are shown at left.

The results described above suggest that glypican-1 is required for the translocation and stabilization of Met to lipid rafts, where it is activated. To test this possibility, cells were treated with MβCD, an antifungal drug that selectively extracts cholesterol from the plasma membrane to disrupt lipid raft structure. WT myoblasts pretreated with MβDC at two different concentrations (1 nM and 10 nM) were stimulated with increasing concentrations of HGF for 5 minutes. Figure 4A shows that total Met levels did not change significantly after treatment, but the HGF-dependent activation of AKT and ERK was diminished in myoblasts with disrupted lipid raft domains. In addition, Figure 4B shows that both Met and caveolin 1 were relocalized from lipid rafts to non-lipid-raft fractions after MβCD treatment.

**Figure 5:**
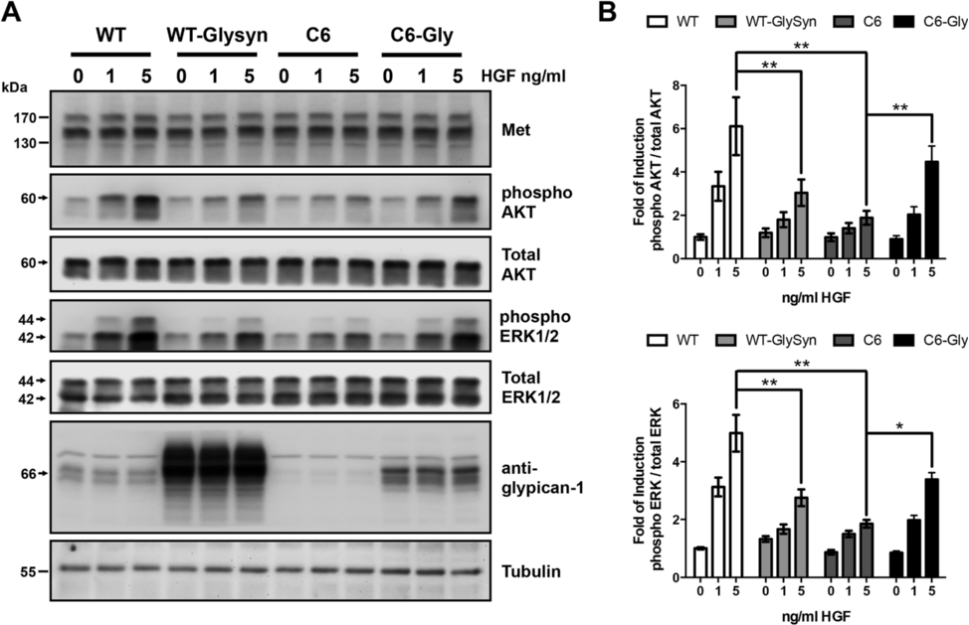
**Glypican-1 is required to sustain the hepatocyte growth factor-dependent signaling in lipid rafts.** Wild-type (WT) myoblasts were transiently transfected with an empty vector as the control or with a non-lipid-raft form of glypican-1 containing the extracellular domain of rat glypican-1 and the transmembrane and cytoplasmic domains of mouse syndecan-1 (F-GlySyn) [36]. C6 myoblasts were transiently transfected with an empty vector as the control or with rat glypican-1 (C6-Gly). Forty-eight hours after transfection, the cells were serum-starved for 6 hours and then treated with the indicated concentrations of hepatocyte growth factor (HGF) for 5 minutes. **(A)** The cell extracts were analyzed by immunoblotting for total HGF receptor (Met), phospho- and total Akt and phosphorylated extracellular signal-regulated kinases 1 and 2 (phospho-ERK1/2) and total ERK1/2. Glypican-1 core protein levels after heparitinase digestion of endogenous and both transfected forms of glypican-1 were detected by using an anti-glypican-1 antibody. Tubulin levels were used as loading controls. **(B)** Quantification from two independent experiments is shown. Statistical significance was assessed using two-way analysis of variance and a Bonferroni multiple-comparisons posttest. *P < 0.05, **P < 0.01.

The results of the present study indicate that Met, phospho-Met and glypican-1 colocalized in lipid raft domains of the plasma membrane. Moreover, glypican-1 expression and lipid raft integrity were required to sustain the HGF-dependent signaling. Next, we evaluated whether glypican-1 *per se* or its presence in lipid raft domains was required to sustain the HGF signaling mediated by the Met receptor. A chimeric form of HSPG containing the extracellular domain of rat glypican-1 and the transmembrane and cytoplasmic domains of mouse syndecan-1 (F-GlySyn) was expressed in WT cells. This chimeric form localized in the non-lipid-raft region of the plasma membrane as we previously reported [38]. Figure 5 shows that mock-transfected WT myoblasts induced the activation of AKT and ERK1/2 in response to HGF. In myoblasts expressing the chimeric F-GlySyn, however, both phospho-AKT and phospho-ERK1/2 levels decreased compared to WT cells. These levels are comparable to levels found in the glypican-1-deficient myoblasts. The figure also shows that diminished sensitivity to HGF, which we had previously observed in the glypican-1-deficient cells, was restored after reexpressing glypican-1 by transient transfection with rat glypican-1. Together, these results indicate that glypican-1 must be associated with lipid rafts to sustain HGF-dependent signaling.

### Glypican-1 physically interacts with HGF and Met in lipid rafts to form an active signaling complex

**Figure 6:**
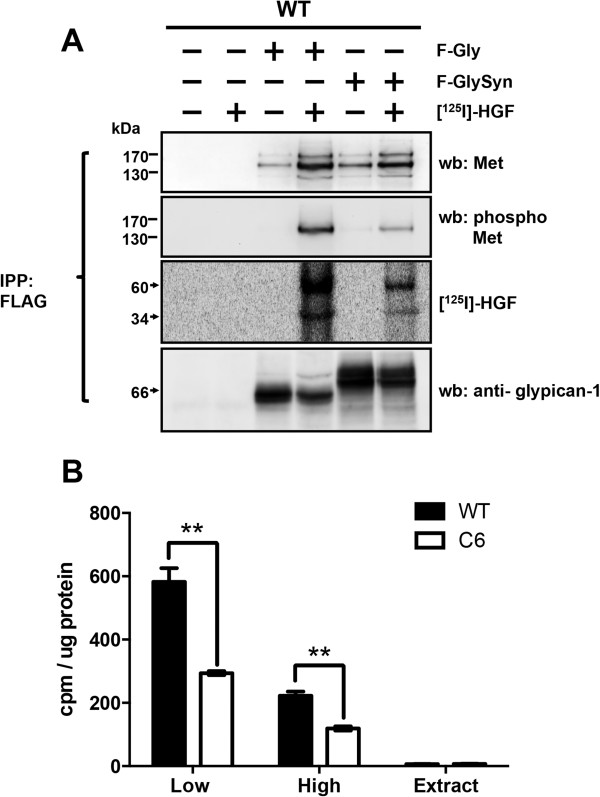
**Glypican-1 in lipid rafts coimmunoprecipitates with the activated form of Met and regulates hepatocyte growth factor binding to low- and high-affinity cell surface binding sites. (A)** C2C12 myoblasts were transfected with rat glypican-1 (F-Gly) or the non-lipid-raft chimeric glypican-1 (F-GlySyn), as described in the Figure 5 legend. F-Gly and F-GlySyn contained a FLAG epitope at the amino terminus. Forty-eight hours after transfection, the cells were serum-starved for 4 hours and then treated with or without 20 ng/ml [^125^I]HGF for 5 minutes. The cell extracts were incubated with anti-FLAG M2 Affinity Gel for 3 hours at 4°C, and, after several washes, the beads were incubated with heparitinase and chondroitinase ABC for 8 hours. The immunoprecipitated (IPP) bound material was eluted with protein loading buffer and analyzed by Western immunoblotting for total hepatocyte growth factor (HGF) receptor (Met), phospho-Met and glypican-1. The membranes were exposed to a phosphorimager to detect [^125^I]HGF. **(B)** C2C12 and C6 myoblasts were serum-starved for 4 hours and then treated with or without 10 ng/ml [^125^I]HGF for 2 hours at 4°C. After several washes in ice-cold binding buffer, [^125^I]HGF was eluted with high salt and acid to determine low- and high-affinity binding sites, respectively. Counts per minute (cpm) were determined by γ counting and corrected for protein content in cell extracts. Statistical significance was assessed by two-way analysis of variance and a Bonferroni multiple comparisons posttest. **P < 0.01.

The results described above suggest that glypican-1 may interact with Met and HGF in lipid rafts to form the ternary complex Met-HGF-glypican-1. To test this possibility, WT myoblasts were transfected with an empty vector as the control or with rat glypican-1 (F-Gly) or chimeric F-GlySyn, both of which contained a FLAG epitope. Forty-eight hours later, the cells were incubated with or without 20 ng/ml [^125^I]HGF for 5 minutes. The cell extracts in the presence of phosphatase inhibitors were immunoprecipitated with anti-FLAG antibodies, and the precipitate was evaluated for total and phospho-Met. Figure 6A shows that, in the absence of HGF, Met coimmunoprecipitated with both F-Gly and F-GlySyn almost to the same extent. When the cells were treated with HGF, the levels of coimmunoprecipitated Met increased with both forms of glypican-1, though in a more pronounced way with F-Gly. Interestingly, when the activated form of precipitated Met was evaluated, F-Gly interacted substantially more than the non-lipid-raft form of glypican-1 (F-GlySyn) with phospho-Met. We also found that [^125^I]HGF coimmunoprecipitated almost four times more with F-Gly than with F-GlySyn. As an immunoprecipitation control, F-Gly and F-GlySyn were detected with specific anti-glypican-1 antibodies. These results suggested that glypican-1 physically interacted with Met and HGF preferentially located in lipid rafts, where the receptor was stabilized and activated in response to HGF. To determine if binding of HGF on the myoblast cell surface was modulated by glypican-1, we performed a ligand binding assay. WT and glypican-1-deficient myoblasts were incubated with [^125^I]HGF at 4°C to avoid endocytosis of the ligand. The radioactivity associated with low- and high-affinity binding sites, as well as the remaining radioactivity in the cell extracts, was determined. Figure 6B shows that the binding of [^125^I]HGF to both low- and high-affinity binding sites was diminished by 50% in the absence of glypican-1, suggesting that this lipid raft–associated HSPG was required to concentrate HGF on the cell surface and for binding to Met. These results indicate that glypican-1 facilitated the binding of HGF to the Met receptor, enhancing its phosphorylation at lipid raft domains.

### Migration of transplanted myoblasts in skeletal muscles is enhanced by HGF and requires glypican-1

**Figure 7:**
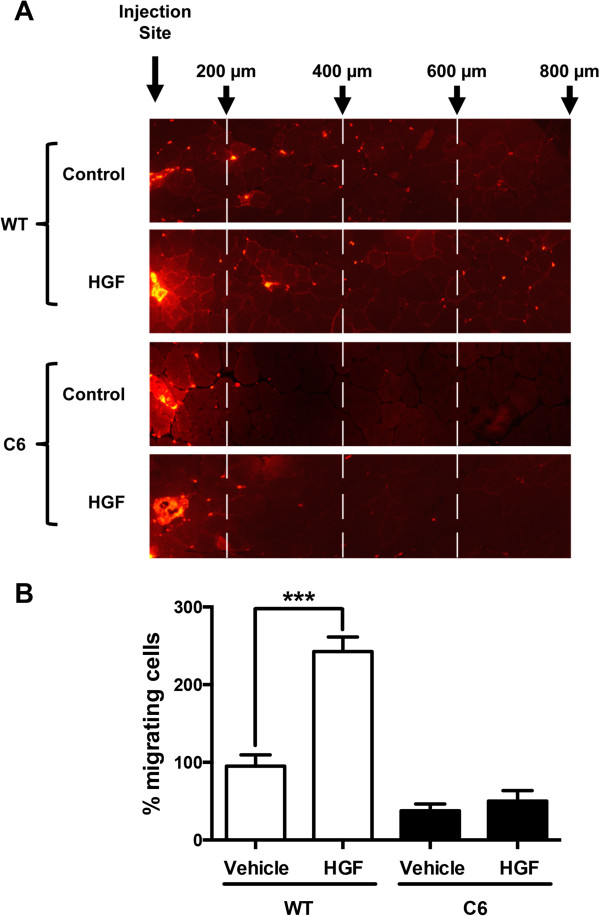
**Hepatocyte growth factor-dependent migration of myoblast *in vivo *requires glypican-1 expression. (A)** Wild-type (WT) or C6 myoblasts (500 × 10^3^), prelabeled with the vital dialkylcarbocyanine dye, DiI (red fluorescence), and suspended in 30 μl of physiological serum with or without 10 ng of carrier-free hepatocyte growth factor (HGF) were transplanted into the left and right tibialis anterior (TA) muscles, respectively, of 3-month-old C57BL/10 mice under anesthesia. One week later, both TA muscles were processed for cryosectioning. Concentric circles with annuli 200 μm from each other were superimposed on selected muscle cross-section images. The cells were counted under an inverted microscope equipped for epifluorescence. **(B)** The number of the cells that migrated more than 200 μm were considered the total migrating cells, and the percentages of cells that migrated more than 600 μm were calculated. The migration of untreated WT myoblasts was corrected to 100%. Values are expressed as mean ± SD of two independent experiments. ***P < 0.001.

The data described above demonstrates the requirement of glypican-1 for HGF-dependent signaling and migration. To test the *in vivo* role of glypican-1 on HGF-induced myoblast migration, we subjected the C57BL/10 mice to intramuscular coinjection of C2C12 or C6 myoblasts together with HGF in the TA muscles. Seven days after the transplantation, the muscles were extracted, frozen in liquid nitrogen and cryosectioned. Prior to grafting, the myoblasts were stained with the vital dialkylcarbocyanine dye, DiI (red fluorescence), to trace their localization in the muscle cryosections. Figure 7 shows that HGF induced an increase in the number of WT myoblasts that migrated longer distances (more than 600 μm). However, this effect was prevented in glypican-1-deficient myoblasts. These results suggest that glypican-1 expression is required for efficient *in vivo* myoblast migration in response to HGF.

## Discussion

One of the main functions of membrane-associated HSPGs, particularly for glypicans, is to regulate signaling of several cytokines, morphogens and growth factors [38,50-53]. It has been reported that loss of HSPG expression prevents the cell mitogenic response induced by HGF [54-56], but the specific roles and mechanisms of the different HSPGs as regulators of HGF-dependent responses have not been studied in depth.

In the present report, we show that, in myoblasts, glypican-1 located in lipid raft membrane domains was required for maximum HGF-dependent signaling and cell migration *in vitro* and *in vivo*. We also show that glypican-1 appears as an essential cell-surface, low-affinity binding site for HGF, likely acting as a presenter or facilitator of HGF to its high-affinity Met binding site, where it is cofractionated with the known HGF coreceptor CD44 [34]. Glypican-1, Met and HGF formed an active signaling ternary complex in lipid raft membrane domains. Whether phospho-Met is relocated from non-lipid-raft to lipid raft domains in response to HGF or whether Met is directly activated in lipid rafts, where it is stabilized, are still not known. Chimeric non-lipid-raft glypican-1 (F-GlySyn) also coimmunoprecipitated with Met, but not with the active form of the receptor or with HGF, indicating that localization of glypican-1 in lipid raft domains was unnecessary for the interaction between Met and the extracellular part of glypican-1, but was required for binding of HGF and subsequent receptor activation.

The participation of lipid rafts as signaling platforms to facilitate interaction of the required elements to activate a signaling pathway has been reported for different receptor tyrosine kinases, such as the platelet-derived growth factor, TrkA/nerve growth factor and insulin receptors. After ligand activation, MAPK and phosphoinositide 3-kinase (PI3K) signaling mediators are recruited to lipid rafts, where they are activated [57-60]. The same mechanism of action has also been reported for G protein–coupled receptors, including β-adrenergic, neurokinin 1 receptor and muscarinic cholinergic receptors [61-64]. Lipid rafts can also act as a platform where receptor signaling is turned off, such as in the case of serine-threonine kinase transforming growth factor β [65] and tyrosine kinase epidermal growth factor receptors, which are activated in lipid rafts, but rapidly relocalized to non–lipid rafts to de-activate downstream signaling [66]. We previously reported that glypican-1 in lipid rafts acted as a negative regulator of FGF-2 signaling, sequestering the growth factor in these domains away from their transducing receptors [38]. Our present results show that, upon ligand binding, Met is recruited to lipid rafts to activate MAPK, ERK1/2 and PI3K/AKT pathways. This process required the presence of structured lipid raft membrane domains as well as glypican-1 in these domains to sustain the HGF-dependent signaling. However, these results did not eliminate the possibility of other Met-dependent functions in non–lipid rafts.

HGF is involved in many different processes in which both cell growth and cell migration are required, such as in embryonic development, tissue repair and organ regeneration [67]. In particular, the roles of HGF and Met for muscle development, differentiation and regeneration have been reported [7]. During limb muscle development, migratory muscle precursor cells delaminate from the dermomyotome, an epithelial structure that develops from somites, reaching their specific destination in the limb buds [68-70] in a process dependent on HGF and Met expression [6-8]. In the present study, we show that glypican-1 was required for the migration of myoblasts in response to HGF, both *in vitro* and *in vivo. In vitro* glypican-1-deficient myoblasts were almost unresponsive to HGF as a chemoattractant in the Boyden chamber assays, in contrast to WT myoblasts, which migrated extensively through the membrane toward the HGF-containing media. The migration capacity toward other chemoattractants did not appear hampered, because no significant differences were observed when both types of cells were challenged to migrate toward 10% FBS (data not shown). We determined the role of glypican-1 in myoblast migration *in vivo* in response to HGF by intramuscular coinjection of WT or glypican-1-deficient myoblasts in the presence or absence of the growth factor. *In vivo* myoblast migration was improved by coinjection with HGF, particularly in WT cells, compared to the slight migratory effect observed with glypican-1-deficient myoblasts. These results show that *in vivo* migration of myoblast expanded *in vitro* could be improved by coinjection with HGF. In addition, this effect required the expression of glypican-1 in the myoblast plasma membrane.

This result is very promising, because one of the main problems associated with stem cell therapies for the treatment of patients with muscular dystrophies is the poor migration of the transplanted cells. As a result, therapy with intramuscular injection of myoblasts or SCs in several clinical trials has been mostly unsuccessful [71-73]. Based upon our results, the use of fluorescence-activated cell sorting with higher expression of glypican-1 and/or coinjection with HGF to improve efficiency needs to be carefully evaluated.

As previously mentioned, HSPGs are essential components required for the myogenic inhibitory signaling of FGF-2 [28,30-33,74-76] and HGF [3,77,78]. During differentiation, expression of all syndecans was downregulated, which is consistent with a reduction in sensitivity to the inhibitory effect of FGF-2 [32,38,79]. In contrast, the expression of glypican-1 remained constant, being the main cell surface HSPG present during myogenesis [35,39]. In addition, during muscle regeneration, expression of glypican-1 increased and was temporarily and histologically related to the newly regenerating myofiber expression of embryonic myosin [80]. However, the exact role of glypican-1 during this process has not been addressed to date. Glypican-1-knockout mice were almost indistinguishable from WT mice in size, fertility, internal anatomy and lifespan, with the exception of the brain, which was noticeably smaller [81]. This suggests that glypican-1 is required in mammals for brain development, but not for other tissues, such as skeletal muscle. To further elucidate the results of the present study, it would be informative to evaluate the skeletal muscle regeneration process in glypican-1-null mice.

Glypican-1 is required for terminal myogenesis, acting as a repressor of FGF-2 [38]. This can be explained by the sequestration of FGF-2 by glypican-1 in lipid rafts, away from FGF-2 receptors and syndecans that are located in non–raft domains. As we have shown, however, glypican-1 positively regulates HGF-mediated signaling by recruiting or stabilizing Met in lipid raft domains where it was activated, with consequential triggering of downstream targets. Reduction of Met expression during the myogenic differentiation process (data not shown) [82] therefore seemed to circumvent the myogenic inhibitory effect of HGF in spite of the constitutive expression of glypican-1 [35,39]. All of these changes switched the balance from a proliferative, migratory and antimyogenic state in response to FGF-2 and HGF to a promyogenic response whereby both muscle inhibitory signals decreased, thus allowing differentiation.

Immediately after injury, low concentrations of HGF (2 to 3 ng/ml) are released from ECM reservoirs [83-85] in conjunction with the local release of nitric oxide. These are the first cues involved in the activation (that is, exit from quiescence) of SCs, which then proliferate to form new fibers or repair the destroyed ones [10,84,86]. To maintain their regenerative potential, many proliferating SCs return to quiescence, repopulating the SC niche to maintain a progenitor pool, which will be activated to repair the muscle in response to a new injury [87-89]. This capacity to repopulate the quiescent SC pool is one of main features of stem cells. It is explained by asymmetric cell division involving some daughter cells, which continue the differentiation pathway, whereas other cells exit the cell cycle and return to quiescence [90,91]. HGF concentrations above 20 ng/ml induced the quiescence of primary myogenic cells. This effect was reversible because treatment with low concentrations of HGF could rescue the proliferation of myogenic cells after high HGF-induced quiescence [77].

It would be interesting to determine whether glypican-1 has a potential role in the control of SC sensitivity to extracellular HGF and to define which SCs will continue to form new muscle and which will exit the cell cycle in asymmetric cell division to maintain the pool of muscle stem cells. Besides its association with lipid rafts in the cell membrane, glypican-1 is also endogenously processed to a soluble form that is incorporated into the ECM [35,38,80], where it can act as a reservoir for HGF and other heparin-binding growth factors that can be released upon an injury to activate SCs. More accurate future studies designed to determine the control mechanisms of glypican-1 and Met expression between daughter cells during asymmetric cell division, as well as the role of glypican-1 during the muscle regeneration process, are therefore necessary.

## Conclusion

Glypican-1 in lipid raft membrane domains is required for maximum HGF-dependent signaling and myoblast migration *in vitro* and *in vivo*.

## Abbreviations

AKT: Effector of the phosphoinositide 3-kinase/AKT pathway; ERK: Extracellular signal-regulated kinase; FGF-2: Fibroblast growth factor 2; FGFR: Transducing fibroblast growth factor receptor; F-Gly: Rat glypican-1 containing a FLAG epitope in its amino terminus; F-GlySyn: Chimeric heparan sulfate proteoglycan (extracellular domain of rat glypican-1 and the transmembrane and cytoplasmic domains of mouse syndecan-1 containing a FLAG epitope); HGF: Hepatocyte growth factor; HS: Heparan sulfate; HSPG: Heparan sulfate proteoglycan; Met: Transducing hepatocyte growth factor receptor; MβCD: Methyl-β-cyclodextrin; Na+/K+-ATPase: Sodium potassium ATP pump; Phospho-AKT: Phosphorylated form of AKT; Phospho-ERK: Phosphorylated form of extracellular signal-regulated kinase; Phospho-Met: Phosphorylated form of hepatocyte growth factor receptor; TA: Tibialis anterior.

## Competing interests

The authors declare that they have no competing interests.

## Authors’ contributions

JG participated in the design of the study, carried out the cellular and molecular experiments, performed the statistical analysis and drafted the manuscript. DC carried out the myoblast migration experiments. EB conceived the study, participated in its design and coordination and helped to draft the manuscript. All authors read and approved the final manuscript.
